# Arterial blood gas parameters as mortality predictors in critically ill patients admitted from the emergency department: a prospective observational cohort study

**DOI:** 10.62838/jccm-2026-0040

**Published:** 2026-10-30

**Authors:** Koca Caliskan, Mehmet Duru

**Affiliations:** Department of Internal Medicine, Division of Intensive Care, Kayseri City Education and Research Hospital, Kayseri, Türkiye; Department of Emergency Medicine, Medical Point Gaziantep Hospital, Gaziantep, Türkiye

**Keywords:** Arterial blood gas, critical illness, ICU severity scores, APACHE, SAPS II, mortality prediction, emergency department, risk stratification

## Abstract

**Background:**

Arterial blood gas (ABG) analysis is rapidly available in emergency and intensive care settings and constitutes a core component of many critical illness scoring systems. Although ABG parameters are incorporated into many multidimensional critical illness scoring systems, their independent contribution to mortality prediction remains uncertain because these variables may change rapidly during the early course of critical illness and resuscitation. This study evaluated the prognostic performance of ABG parameters and validated severity scoring systems for predicting 28-day mortality in critically ill patients admitted from the emergency department (ED) to the intensive care unit (ICU).

**Methods:**

In this prospective observational cohort study, 242 adult patients admitted from the ED to the internal medicine ICU between January 2012 and March 2013 were enrolled. Admission ABG parameters (pH, PaCO_2_, PaO_2_, and HCO_3_^−^) and severity scores (APACHE II, APACHE IV, and SAPS II) were recorded. The primary outcome was 28-day all-cause mortality. Prognostic discrimination was assessed using receiver operating characteristic (ROC) curve analysis.

**Results:**

Overall 28-day mortality was 47.1%. Non-survivors had significantly higher APACHE II, APACHE IV, and SAPS II scores than survivors (all p < 0.001). SAPS II demonstrated the best prognostic performance (AUC 0.720; 95% CI 0.659–0.776), significantly outperforming APACHE II and APACHE IV. In contrast, none of the individual ABG parameters independently predicted 28-day mortality (all p > 0.05).

**Conclusions:**

Multidimensional severity scoring systems, particularly SAPS II, provide superior prognostic discrimination compared with isolated ABG parameters in critically ill ED patients. Although ABG analysis remains essential for acute physiologic assessment, isolated blood gas variables appear insufficient as standalone mortality predictors in heterogeneous ICU populations.

## Introduction

Emergency department (ED) overcrowding represents an escalating global public health problem, imposing substantial strain on hospital resources and compromising the timely care of critically ill patients [1, 2]. Within this constrained environment, identifying patients who require immediate ICU admission and intensive monitoring is paramount. Failure to recognize deteriorating patients early is associated with increased adverse outcomes, delayed intervention, and avoidable mortality [3]. Conversely, unnecessary ICU admissions consume limited resources and expose stable patients to unnecessary risks. Robust, early prognostic stratification tools are therefore essential for both individual patient management and system-level resource allocation.

Arterial blood gas (ABG) analysis is one of the most rapidly available investigations in emergency and critical care medicine, routinely obtained within minutes of patient assessment. It provides immediate data on oxygenation, ventilation, and acid-base status, and is integrated into virtually every acute physiologic scoring system. As such, ABG parameters are often intuitively applied by emergency and critical care clinicians as prognostic surrogates. Specifically, severe acidemia, hypercapnia, hypoxemia, and markedly reduced bicarbonate concentrations are commonly interpreted as indicators of grave prognosis [4, 5]. This clinical heuristic is physiologically plausible: profound metabolic derangements reflect tissue hypoperfusion, organ dysfunction, and the failure of compensatory mechanisms.

However, the prognostic value of isolated pretreatment ABG variables—considered independently rather than as components of composite scores—remains incompletely characterized, particularly in heterogeneous internal medicine ICU populations presenting from the ED. Critically ill patients admitted from the ED encompass an extraordinarily diverse case mix: respiratory failure, sepsis, acute renal failure, neurological emergencies, toxicological presentations, metabolic crises, and cardiac arrest, among others. In this context, a single metabolic parameter measured at a single time point is subject to numerous confounders, including the timing and degree of early resuscitation, compensatory physiologic responses, chronic comorbidities, and mixed pathophysiologic states [6, 7]. Recent critical care research has increasingly emphasized the limitations of static single-time-point prognostic models and highlighted the importance of dynamic physiologic monitoring and continuously updated risk prediction systems in critically ill populations [8, 9].

Multidimensional severity scoring systems—including the Acute Physiology and Chronic Health Evaluation (APACHE) II and IV and the Simplified Acute Physiology Score (SAPS) II—were developed precisely to address this complexity. By integrating multiple physiologic variables with age and chronic health status, these instruments provide a broader characterization of disease burden and have demonstrated superior discriminative ability for ICU mortality in validated cohorts [10, 11]. Nevertheless, these scoring systems are predominantly validated in established ICU populations rather than in patients at the point of ED-to-ICU transfer, and their comparative performance against simple ABG parameters in this specific transition zone is not uniformly established.

This study prospectively evaluated critically ill adult patients admitted from the ED to the internal medicine ICU, aiming to determine whether pretreatment ABG parameters—pH, PaCO_2_, PaO_2_, and HCO_3_^−^—independently predict 28-day mortality and whether multidimensional scoring systems (APACHE II, APACHE IV, SAPS II) provide superior prognostic discrimination.

## Methods

This prospective observational cohort study was conducted in the Emergency Department of Mustafa Kemal University Tayfur Ata Sökmen Training and Research Hospital between January 2012 and March 2013. The study was performed in accordance with the principles of the Declaration of Helsinki. Written informed consent was obtained from all participants or, in cases of impaired decision-making capacity, from their legally authorized representatives. Ethical approval was granted by the Clinical Research Ethics Committee of Mustafa Kemal University Faculty of Medicine (Approval No: 2012/302). The study was also published in thesis format in the National Thesis Center of the Council of Higher Education under the title “The Importance of Blood Gas Assessment in the Management of Critically Ill Patients and in Determining Mortality, Intensive Care Requirement, and Length of ICU Stay” (Thesis No: 443770).

To evaluate the prognostic value of arterial blood gas parameters for 28-day mortality, an a priori power analysis indicated that 220 subjects would provide 80% power to detect an AUC of 0.60, compared to a baseline AUC of 0.50, with a two-tailed alpha level of 0.05 and a predicted event rate of nearly 50%. Given that the present study enrolled 242 patients, the cohort possessed adequate statistical power to discern modest ROC-based predictive capacities. Nonetheless, identifying marginal prognostic contributions from specific blood gas parameters remains constrained, requiring expansive multicenter cohorts for validation.

A post hoc power analysis was performed for the primary ROC-based prognostic analysis of 28-day mortality. Using the observed sample size of 242 patients, including 114 non-survivors and 128 survivors, and the observed AUC of SAPS II for 28-day mortality prediction (AUC = 0.720), the achieved statistical power was greater than 99.9% at a two-sided alpha level of 0.05. The corresponding achieved power values were 99.6% for APACHE II (AUC = 0.661) and 99.1% for APACHE IV (AUC = 0.653). These findings indicate that the final cohort was adequately powered to detect the observed discriminatory performance of the multidimensional severity scoring systems. However, smaller discriminatory effects of individual arterial blood gas parameters would require larger cohorts.

From a consecutive screening population of 774 patients admitted from the ED to the internal medicine ICU during the study period, 242 patients were enrolled. Inclusion criteria were: (1) adult age (≥18 years); (2) arterial blood gas sampling performed in the ED prior to ICU admission; (3) provision of informed consent. Exclusion criteria were: (1) age <18 years; (2) pregnancy; (3) primary traumatic injury; (4) requirement for surgical intervention as the primary management strategy; (5) incomplete data precluding full scoring system calculation. Patients for whom specific variables essential to scoring calculations were unavailable solely due to absent clinical indication (i.e., not omitted as a result of the study protocol) were excluded. A detailed flow diagram illustrating patient screening, exclusion criteria, follow-up procedures, and final cohort allocation is presented in Figure 1. Missing data were handled by complete-case analysis. Patients with incomplete baseline data preventing calculation of APACHE II, APACHE IV, or SAPS II scores were excluded before study inclusion. No imputation procedures were performed. Patients with unavailable 28-day outcome information were excluded during the screening process, as shown in Figure 1. Therefore, the final analytical cohort consisted only of patients with complete baseline variables required for severity score calculation and confirmed 28-day vital status.

**Fig. 1.**
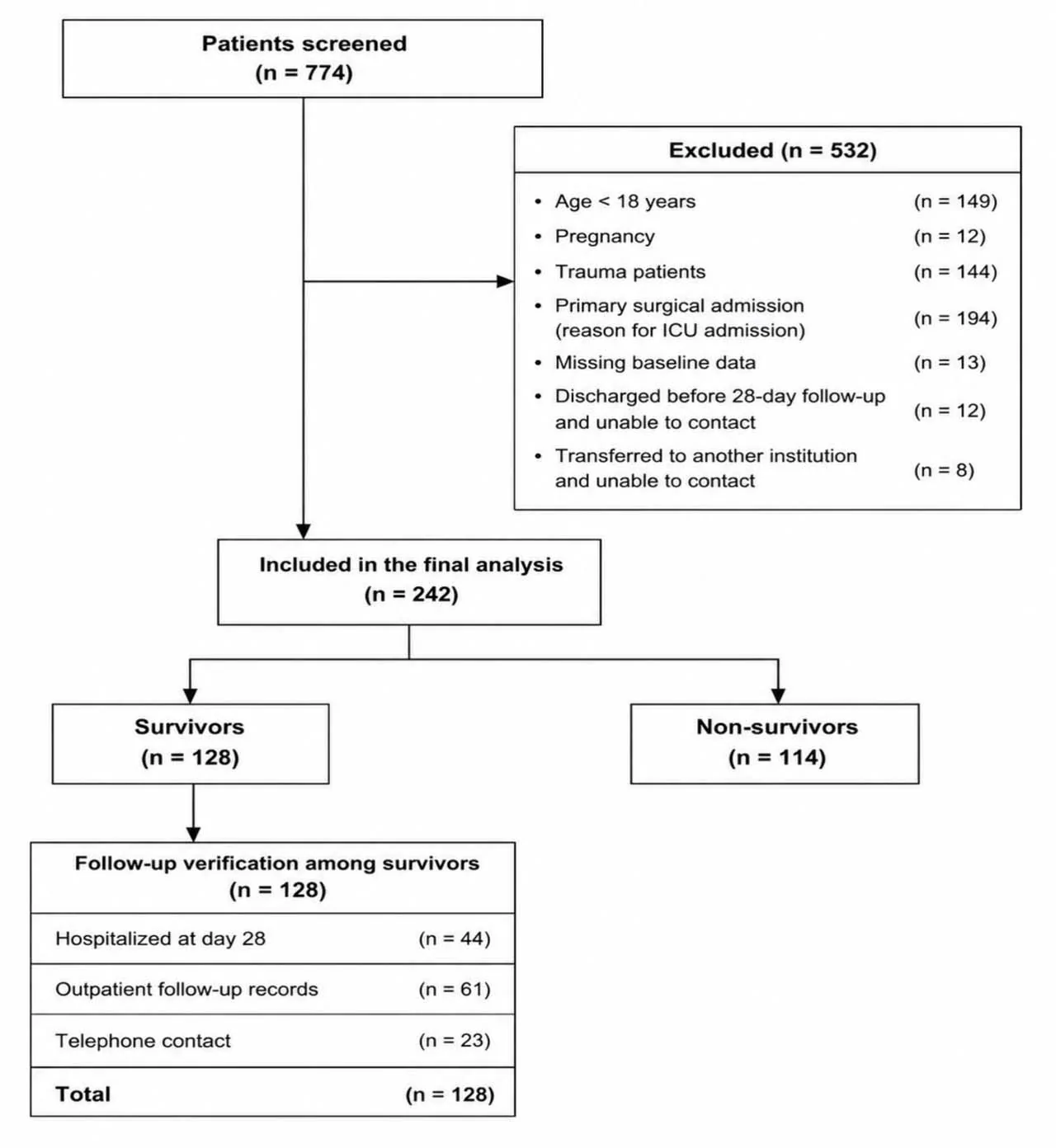
Study Flow Diagram Showing Patient Screening, Exclusion Criteria, Follow-up Procedures, and Final Cohort Allocation

Demographic, clinical, and laboratory data were collected prospectively. ABG parameters—pH, PaCO_2_ (mmHg), PaO_2_ (mmHg), and HCO_3_^−^ (mEq/L)—were measured from samples obtained in the ED using a point-of-care blood gas analyzer prior to ICU transfer and during the initial ED evaluation. APACHE II, APACHE IV, and SAPS II scores were calculated using the most abnormal values recorded during the emergency department stay and early ICU admission. For variables requiring 24-hour assessment windows, the worst available physiologic values documented during this period were used for score calculation. Glasgow Coma Scale (GCS) scores were recorded prior to any sedation; pre-sedation scores were used where applicable.

The primary outcome was all-cause 28-day mortality. All patients were followed for a minimum of 28 days. Vital status was determined using hospital records, outpatient follow-up documentation, and direct telephone contact when necessary.

Categorical variables are presented as frequencies and percentages; continuous variables as means ± standard deviation (SD). Between-group comparisons of continuous variables were performed using the Student's independent t-test. Categorical associations were assessed with the chi-square test. Receiver operating characteristic (ROC) curve analysis was performed to evaluate the discriminatory performance of each scoring system and ABG parameter for 28-day mortality, with results reported as area under the curve (AUC) with 95% confidence intervals (CI). Pairwise AUC comparisons were performed using the DeLong method. Survival distributions were estimated by the Kaplan-Meier method and compared between groups using the log-rank test. Correlation between scoring systems and between bicarbonate and base deficit was assessed with linear regression. All analyses were performed using SPSS version 20.0 (IBM Corporation, Armonk, NY, USA) and MedCalc Statistical Software. A two-sided p-value < 0.05 was considered statistically significant.

## Results

A total of 242 patients were enrolled (129 male [53.3%], 113 female [46.7%]). Overall 28-day mortality was 47.1% (n = 114). Mean age of the entire cohort was 62.95 years (median 67 years). Non-survivors were significantly older than survivors (mean age 67.86 vs. 58.57 years; p < 0.05).

The primary admission diagnoses were: respiratory failure (n = 86; 35.5%), acute renal failure (n = 42; 17.4%), neurological emergencies (n = 29; 12.0%), gastrointestinal/hepatic causes (n = 18; 7.4%), cardiac arrest (n = 20; 8.3%), hematological disorders (n = 14; 5.8%), toxicological presentations (n = 13; 5.4%), endocrine emergencies (n = 12; 5.0%), and sepsis (n = 8; 3.3%). Mortality varied substantially by diagnostic category: the highest mortality was observed in cardiac arrest patients (90%), while endocrine emergencies (0%) and toxicological presentations (7.7%) had the lowest mortality rates (p < 0.001). Toxicological and hematological patients were significantly younger than other diagnostic subgroups (p < 0.05). Baseline demographic and clinical characteristics of the study population are presented in Table 1, while the distribution of diagnostic categories is summarized in Table 2.

**Table 1:** Baseline Characteristics by Outcome

**Variable**	**Survivors (n=128)**	**Non-survivors (n=114)**	**p-value**
Age, years (mean ± SD)	58.57	67.86	<0.001
Male sex, n (%)	76 (59.4%)	53 (46.5%)	0.052
APACHE II score (mean)	16.2	21.1	<0.001
APACHE IV score (mean)	53.6	66.5	<0.001
SAPS II score (mean)	32.1	45.3	<0.001

SD = standard deviation. APACHE = Acute Physiology and Chronic Health Evaluation; SAPS = Simplified Acute Physiology Score.

**Table 2:** Diagnostic Category Distribution and 28-Day Mortality

**Diagnostic Category**	**Total (n)**	**Survivors**	**Deaths**	**Mortality (%)**
Respiratory failure	86	46	40	46.5
Acute renal failure	42	24	18	42.9
Neurological	29	8	21	72.4
Cardiac arrest	20	2	18	90.0
Gastrointestinal	18	12	6	33.3
Hematologic	14	10	4	28.6
Toxicology	13	12	1	7.7
Endocrine	12	12	0	0.0
Sepsis	8	2	6	75.0
Total	242	128	114	47.1

Chi-square test across diagnostic categories: p<0.001.

All three scoring systems were significantly associated with 28-day mortality. Mean APACHE II scores were 16.2 ± 7.4 in survivors versus 21.1 ± 8.5 in non-survivors (p < 0.001). Mean APACHE IV scores were 53.6 in survivors versus 66.5 in non-survivors (p < 0.001). Mean SAPS II scores were 32.1 in survivors versus 45.3 in non-survivors (p < 0.001). All three systems were mutually correlated by linear regression (all p < 0.001).

On ROC analysis, SAPS II demonstrated the highest discriminatory performance for 28-day mortality (AUC = 0.720; 95% CI 0.659–0.776), followed by APACHE II (AUC = 0.661; 95% CI 0.598–0.721) and APACHE IV (AUC = 0.653; 95% CI 0.589–0.712). Pairwise comparison demonstrated that SAPS II significantly outperformed both APACHE II (difference in AUC = 0.059; p = 0.022) and APACHE IV (difference in AUC = 0.067; p = 0.006). The difference between APACHE II and APACHE IV was not statistically significant (AUC difference = 0.009; p = 0.756). A summary of the ROC curve analyses is provided in Table 3, and the corresponding ROC curves are illustrated in Figure 2.

**Fig. 2.**
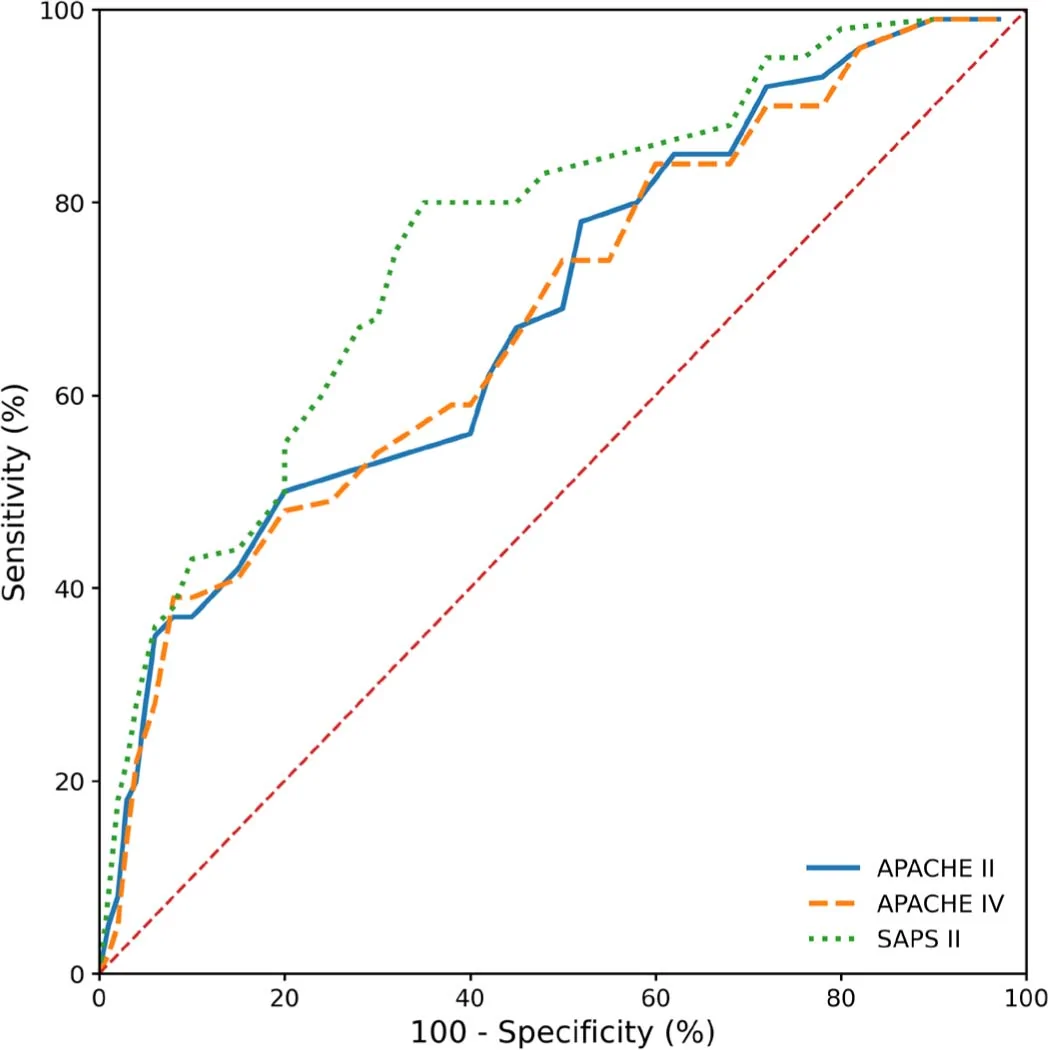
Receiver Operating Characteristic (ROC) Curves for Severity Scoring Systems Receiver operating characteristic (ROC) curves for APACHE II (solid blue line), APACHE IV (dashed orange line), and SAPS II (dotted green line) for prediction of 28-day all-cause mortality in 242 critically ill patients admitted from the emergency department to the intensive care unit. SAPS II demonstrated the highest discriminatory performance (AUC 0.720; 95% CI 0.659–0.776), significantly outperforming APACHE II (AUC 0.661; p = 0.022) and APACHE IV (AUC 0.653; p = 0.006). The diagonal reference line represents chance discrimination (AUC = 0.500). P-values were calculated using DeLong pairwise comparisons.

**Table 3:** ROC Curve Analysis: Severity Scoring Systems for 28-Day Mortality Prediction

**Scoring System**	**AUC**	**95% CI**	**SE**	**p-value**
SAPS II	0.720	0.659–0.776	0.0326	<0.001
APACHE II	0.661	0.598–0.721	0.0348	<0.001
APACHE IV	0.653	0.589–0.712	0.0350	<0.001
Pairwise comparisons (DeLong method):				
Comparison	AUC Difference	95% CI		p-value
SAPS II vs. APACHE II	0.059	0.008–0.109		0.022
SAPS II vs. APACHE IV	0.067	0.020–0.115		0.006
APACHE II vs. APACHE IV	0.009	−0.047 to 0.065		0.756

AUC = area under the ROC curve; CI = confidence interval; SE = standard error; SAPS = Simplified Acute Physiology Score; APACHE = Acute Physiology and Chronic Health Evaluation.

ICU length of stay was significantly correlated with all three scoring systems (APACHE II and IV: p < 0.001; SAPS II: p = 0.01). However, substantial scatter in regression plots indicated that individual-level LOS prediction from these scores should be interpreted with caution.

Of the 242 patients, 19.4% (n = 47) had a normal blood gas profile; 26.7% (n = 67) had mixed acid-base disturbances. The overall mortality of patients with a normal ABG at ED presentation was 55.3%, exceeding the overall cohort mortality rate—underscoring that a normal initial blood gas does not exclude critical illness.

When stratified by pH category (severe acidosis pH ≤ 7.20; acidosis pH 7.21–7.34; normal pH 7.35–7.45; alkalosis pH ≥ 7.46), 28-day mortality rates were 50.0%, 46.4%, 51.7%, and 33.3%, respectively. Differences across pH groups were not statistically significant (chi-square p = 0.300). Kaplan-Meier survival analysis showed no significant difference in survival curves across pH groups (log-rank p = 0.118). ROC analysis confirmed that pH was not a significant discriminator of 28-day mortality (AUC p = 0.532). pH was not correlated with ICU length of stay (p = 0.050). The distribution of arterial blood gas profiles and the association between admission pH categories and 28-day mortality are presented in Tables 4 and 5, respectively.

**Table 4:** Distribution of arterial blood gas profiles and associated 28-day mortality

**Arterial blood gas profile**	**Total, n (%)**	**Death, n**	**Discharged, n**	**Mortality (%)**
Metabolic acidosis	37 (15.3)	13	24	35.1
Metabolic alkalosis	12 (5.0)	8	4	66.7
Metabolic alkalosis + respiratory alkalosis	2 (0.8)	0	2	0.0
Normal arterial blood gas profile	47 (19.4)	26	21	55.3
Respiratory acidosis	43 (17.8)	22	21	51.2
Respiratory acidosis + metabolic acidosis	43 (17.8)	23	20	53.5
Respiratory acidosis + metabolic alkalosis	8 (3.3)	6	2	75.0
Respiratory alkalosis	36 (14.9)	10	26	27.8
Respiratory alkalosis + metabolic acidosis	14 (5.8)	6	8	42.9
Total	242 (100)	114	128	47.1

**Table 5:** Association between admission pH categories and 28-day mortality

**pH category**	**Definition**	**Total, n (%)**	**Death, n**	**Discharged, n**	**Mortality (%)**
Severe acidosis	pH ≤ 7.20	48 (19.8)	24	24	50.0
Acidosis	pH 7.21–7.34	69 (28.5)	32	37	46.4
Normal pH	pH 7.35–7.45	89 (36.8)	46	43	51.7
Alkalosis	pH ≥ 7.46	36 (14.9)	12	24	33.3
Total		242 (100)	114	128	47.1

Statistical analysis: χ^2^ = 3.666, df = 3, p = 0.300.

Serum HCO_3_^−^ was grouped according to SAPS II-analogous thresholds (< 15, 15–19, ≥ 20 mEq/L). Overall chi-square analysis suggested a significant difference across groups (p = 0.023); however, post-hoc pairwise analysis revealed no statistically significant difference between HCO_3_^−^ < 15 and HCO_3_^−^ ≥ 20 groups (p = 0.586), indicating the association was non-monotonic and not consistent with independent prognostic utility. ROC analysis confirmed that HCO_3_^−^ was not an independent mortality predictor (AUC = 0.540; p = 0.276). A strong linear correlation was observed between HCO_3_^−^ and base deficit/excess (regression coefficient r = 0.879; p < 0.001).

PaCO_2_ did not differ significantly between survivors and non-survivors (38.7 vs. 43.0 mmHg; p = 0.067). When stratified by hypocapnia (PaCO_2_ < 35 mmHg), normocapnia (35–45 mmHg), and hypercapnia (> 45 mmHg), mortality rates were 35.9%, 58.2%, and 57.1%, respectively (chi-square p = 0.003); however, Kaplan-Meier analysis demonstrated no significant survival difference across these groups (log-rank p = 0.223). PaCO_2_ was not an independent mortality predictor.

PaO_2_ did not differ significantly between survivors and non-survivors (87.7 ± 49.6 vs. 82.0 ± 55.0 mmHg; p = 0.402). No independent prognostic association between PaO_2_ and 28-day mortality was identified.

The results of the univariable and multivariable logistic regression analyses are presented in Table 6. In univariable analysis, admission arterial blood gas parameters, including pH (p = 0.335), HCO_3_^−^ (p = 0.286), PaCO_2_ (p = 0.128), and PaO_2_ (p = 0.440), were not significantly associated with 28-day mortality. In contrast, established severity scoring systems demonstrated significant prognostic associations with mortality, including SAPS II (p < 0.001), APACHE II (p < 0.001), and APACHE IV (p < 0.001). In the multivariable logistic regression model adjusted for SAPS II, none of the arterial blood gas parameters remained independently associated with 28-day mortality, including pH (p = 0.103), HCO_3_^−^ (p = 0.433), PaCO_2_ (p = 0.276), and PaO_2_ (p = 0.280). SAPS II remained the only independent predictor of mortality in the adjusted model (p < 0.001). The final multivariable model demonstrated acceptable prognostic performance, with a Nagelkerke R^2^ value of 0.265 and an overall classification accuracy of 68.2%, further supporting the superior prognostic utility of severity scoring systems compared with isolated arterial blood gas parameters.

**Table 6:** Univariable and multivariable logistic regression analyses for predictors of 28-day mortality

**Variable**	**Univariable analysis**			**Multivariable analysis**		
	OR	p-value	95% CI	OR	p-value	95% CI
pH	0.484	0.335	0.111–2.111	0.250	0.103	0.047–1.319
HCO_3_^−^	1.013	0.286	0.989–1.038	1.011	0.433	0.978–1.045
PaCO_2_	1.009	0.128	0.998–1.021	1.007	0.276	0.995–1.019
PaO_2_	0.998	0.440	0.992–1.003	0.997	0.280	0.992–1.003
SAPS II	1.069	<0.001	1.046–1.093	1.070	<0.001	1.046–1.095
APACHE II	1.075	<0.001	1.040–1.112	—	—	—
APACHE IV	1.037	<0.001	1.021–1.053	—	—	—

SAPS = Simplified Acute Physiology Score; APACHE = Acute Physiology and Chronic Health Evaluation.

## Discussion

This prospective cohort study of 242 critically ill internal medicine patients admitted from the ED to the ICU demonstrates two principal findings. First, multidimensional severity scoring systems—especially SAPS II—significantly outperform individual ABG parameters in discriminating 28-day mortality, supporting the primacy of integrated physiologic assessment over isolated metabolic variables in this setting. Second, none of the pretreatment ABG parameters examined (pH, PaCO_2_, PaO_2_, HCO_3_^−^) functioned as independent mortality predictors, despite the high physiologic plausibility of such associations. These findings have important implications for how early prognostic information should be interpreted and communicated in emergency and critical care medicine.

The superior discriminatory performance of SAPS II (AUC = 0.720) over APACHE II (AUC = 0.661) and APACHE IV (AUC = 0.653) in this study is consistent with several contemporary comparative analyses. SAPS II was originally developed and validated across a large multinational European-North American cohort, demonstrating robust performance across diverse ICU case mixes [12]. Its relative efficiency—requiring fewer and simpler variables than APACHE IV—may render it more practical for rapid bedside application in the ED context, where resource constraints and time pressure are inherent. Our findings align with Kellner et al., who similarly identified SAPS II superiority over APACHE II and III in cardiogenic shock complicating acute myocardial infarction [13]. In contrast, Vasilevskis et al., in a large multicenter analysis, found APACHE IV superior to SAPS II for ICU mortality, potentially reflecting population-specific differences and the additional predictors incorporated in APACHE IV [14]. The absence of a universally dominant scoring system highlights the importance of population context and the need for local validation.

The fundamental reason multidimensional scoring systems outperform isolated biomarkers is conceptual: they capture the cumulative physiologic burden of illness across multiple organ systems simultaneously, integrating cardiovascular, neurological, respiratory, renal, and metabolic parameters alongside age and comorbid conditions [15]. This composite representation more accurately reflects the totality of physiologic insult than any single variable can. Modern risk stratification research in sepsis, acute respiratory distress syndrome (ARDS), and acute kidney injury has consistently demonstrated that outcome prediction improves with integration of multiple variables, and that single-biomarker approaches—whether metabolic, inflammatory, or hemodynamic—have limited independent discriminatory power [16, 17].

The absence of independent prognostic significance for pH, PaCO_2_, PaO_2_, and HCO_3_^−^ in this study warrants careful interpretation and should not be conflated with clinical unimportance. ABG analysis remains indispensable for immediate physiologic assessment, ventilatory management, acid-base correction, and oxygen delivery optimization [18]. Rather, these findings indicate that single-time-point ABG values, obtained at ED presentation and prior to treatment initiation, lack the independent discriminatory power to predict 28-day mortality across a heterogeneous critically ill population.

Several mechanistic factors explain this limitation. First, ABG values at ED presentation represent a single snapshot in a highly dynamic physiologic process. The trajectory of metabolic derangement—whether improving or worsening—carries far more prognostic information than the absolute value at any single time point. Contemporary ICU literature has progressively shifted toward dynamic prognostic markers, lactate clearance being the most established example [19, 20]. A single lactate or pH measurement on admission reflects the physiologic state at that instant; serial measurements capturing the response to resuscitation provide substantially more prognostic information [21]. Recent studies and scoping reviews have increasingly focused on dynamic and continuously updated ICU prediction models, emphasizing that longitudinal physiologic trends may provide superior prognostic discrimination compared with static admission measurements alone [8, 9, 22].

Second, the critically ill population is characterized by pathophysiologic heterogeneity. Severe acidemia may reflect reversible, rapidly correctable conditions (e.g., diabetic ketoacidosis with appropriate insulin therapy, hyperchloremic acidosis from saline resuscitation) as readily as it may reflect irreversible multi-organ failure. Conversely, patients with preserved normal ABG values—19.4% of our cohort—had a mortality rate of 55.3%, exceeding the overall cohort average. This counterintuitive observation illustrates that normal initial blood gas does not exclude critical illness severity, particularly in patients with mixed or subclinical presentations, those with advanced chronic comorbidities with blunted physiologic reserve, or those already partially compensated on presentation. This finding underscores the fundamental inadequacy of any single parameter to capture prognosis in heterogeneous populations.

Third, physiologic compensatory mechanisms confound the relationship between single ABG variables and outcomes. pH, in particular, integrates both the primary disorder and the degree of compensation: a patient with metabolic acidosis and an appropriate respiratory alkalotic response may have a near-normal pH despite significant tissue dysoxia, while another patient with limited ventilatory reserve may manifest a profoundly abnormal pH despite a less severe primary insult. These compensatory interactions render single-variable interpretations unreliable as independent prognostic tools [23]. Our finding that normal pH groups showed similar or higher mortality than certain acidotic groups exemplifies this complexity.

Fourth, early resuscitative interventions initiated in the ED—intravenous fluids, bicarbonate administration, supplemental oxygen, ventilatory support, vasopressors—begin to alter ABG values within minutes of assessment. The ABG obtained “pretreatment” in a busy ED is inevitably obtained at variable stages of resuscitation, introducing systematic measurement variability that degrades the signal-to-noise ratio of any single value [24]. This timing variability is inherent to the prospective observational design and cannot be fully controlled without introducing artificial delays in care.

The non-significance of pH as an independent mortality predictor in this study is consistent with data from other heterogeneous critical care populations. Minana et al. found no association between admission arterial pH and mortality in acute decompensated heart failure [25], and Kaplan and Kellum similarly reported no significant pH-based mortality discrimination in trauma patients [26]. However, disease-specific studies have yielded different conclusions: Afessa et al. observed pH differences between survivors and non-survivors in a COPD-specific cohort [27], and Burri et al. identified pH as an independent predictor of 12-month mortality in patients presenting with acute dyspnea, though not 30-day mortality [4]. These discordant findings reinforce that the prognostic utility of pH is context-dependent and population-specific, rather than universally applicable across the diverse internal medicine ICU case mix.

The finding that HCO_3_^−^ was not an independent mortality predictor, despite a significant chi-square across grouped categories, reflects the heterogeneity of conditions producing low bicarbonate. Severely reduced HCO_3_^−^ may reflect acute metabolic acidosis from septic shock or lactic acidosis, or it may reflect chronic respiratory alkalosis with compensatory bicarbonate wasting. It may also be elevated in the context of respiratory acidosis compensation, rendering low values falsely reassuring or elevated values falsely concerning depending on the underlying mechanism. Surbatovic et al. reported that serum bicarbonate was a weak mortality predictor (AUC = 0.640 in a surgical ICU population), consistent with our findings, and that it primarily reflected metabolic acidosis severity rather than overall disease burden [28].

The observation that hypocapnic patients (PaCO_2_ < 35 mmHg) had lower cross-sectional mortality rates than normo- or hypercapnic patients, yet did not demonstrate significantly different survival curves on Kaplan-Meier analysis, illustrates the perils of single-time-point cross-sectional comparisons. Hypocapnia may reflect compensatory hyperventilation in response to metabolic acidosis or systemic hypoxemia—a physiologically appropriate and potentially protective response in the early stages of illness—and thus does not carry the same prognostic weight as primary hypercapnia from respiratory failure [29]. Specific ICU populations (post-cardiac arrest, community-acquired pneumonia) have shown bidirectional associations between PaCO_2_ and mortality, further illustrating the population-specificity of these relationships [30, 31].

It is important to contextualize these findings within the modern conceptual framework of sepsis and critical illness physiology. Elevated lactate and acid-base disturbances are well-established markers of physiologic stress and inadequate oxygen delivery; they are integral to early warning systems and sepsis management bundles [32, 33]. The Surviving Sepsis Campaign and multiple international guidelines emphasize lactate-guided resuscitation as a cornerstone of early sepsis management precisely because elevated lactate at presentation identifies patients at risk who require early, aggressive intervention [34]. However, the prognostic role of early lactate—and by extension, acid-base parameters—is primarily as a resuscitation target and early warning signal, not as a definitive independent predictor of final outcome. The absolute degree of initial derangement does not necessarily determine outcome; the response to treatment does [35]. This distinction between acute metabolic derangement and the global burden of organ dysfunction is critical and aligns precisely with our finding that composite scoring systems, which encode the depth of multi-organ dysfunction, outperform point-in-time metabolic parameters.

These findings carry direct practical implications for emergency and critical care clinicians. ABG analysis should not be abandoned or de-emphasized; it remains irreplaceable for immediate clinical decisions regarding ventilatory management, acid-base correction, oxygen titration, and resuscitation monitoring. However, clinicians should be cautious about using isolated ABG values—particularly a single early pH or PaCO_2_—as the primary basis for prognostic communication to patients and families, or as the sole trigger for escalation or withdrawal of care decisions. Such decisions demand the richer context provided by multidimensional severity assessment.

The practical superiority of SAPS II, with its lower variable burden and rapid calculability (approximately 5 minutes at the bedside), makes it particularly well-suited for the ED-ICU interface—an environment where clinicians must rapidly synthesize complex information under time pressure. Deploying SAPS II systematically at the point of ICU admission from the ED could support triage decisions, communication of prognosis to families, and allocation of high-intensity monitoring resources. These applications should be calibrated with the knowledge that SAPS II, even as the best-performing system in our analysis, achieved only moderate discrimination (AUC = 0.720), reinforcing that no current scoring system alone provides sufficient certainty for high-stakes individual clinical decisions.

This study's strengths include its prospective design, minimum 28-day follow-up with active patient tracing, and inclusion of a diverse internal medicine ICU case mix that reflects the real-world heterogeneity of ED-admitted critically ill patients. The simultaneous comparison of three validated severity scoring systems with multiple ABG parameters across a common population provides a direct and clinically relevant head-to-head comparison.

Several important limitations must be acknowledged. First, the single-center design conducted at one academic institution may limit generalizability to settings with different patient demographics, disease epidemiology, and ICU practices. Second, the cohort size (n = 242) is moderate, and while adequately powered for the primary analysis, subgroup analyses by specific diagnostic category are limited by small cell sizes. Third, the study was conducted between 2012 and 2013; the contemporary critical care landscape—including the implementation of sepsis-3 definitions, lung-protective ventilation protocols, and expanded point-of-care diagnostics—may have evolved since that period. However, the fundamental physiologic relationships examined are unlikely to have changed substantively. Fourth, serial ABG measurements were not available, precluding assessment of dynamic changes in acid-base parameters, which may carry greater prognostic utility than single time-point values. Fifth, this study specifically focused on conventional ABG-derived physiologic variables commonly incorporated into classical severity scoring systems. Lactate was intentionally not included because it represents a distinct metabolic biomarker with different pathophysiologic and prognostic characteristics. Sixth, severity scores were calculated based on ED data and early ICU values; the timing variability of score calculation relative to treatment initiation may introduce measurement heterogeneity. Finally, the study population excluded surgical and trauma patients, limiting applicability to these groups.

## Conclusion

In this prospective cohort study involving critically ill internal medicine patients admitted to the ICU from the emergency department, multidimensional severity scoring systems—particularly SAPS II—demonstrated significantly superior performance in predicting 28-day mortality compared with individual arterial blood gas (ABG) parameters. ABG components such as pH, PaCO_2_, PaO_2_, and HCO_3_^−^ provide rapid physiological assessment during the initial evaluation of critically ill patients and remain indispensable in acute intensive care management. However, these parameters may change substantially over short periods in response to therapeutic interventions, ventilatory strategies, fluid resuscitation, and the dynamic clinical course of critical illness.

Therefore, although arterial blood gas parameters constitute central components of many prognostic scoring systems, they may not adequately reflect the true severity of critical illness or accurately predict mortality risk when interpreted in isolation. The rapidly reversible or transiently deteriorating nature of these variables may lead to underestimation or overestimation of disease severity in some patients, thereby reducing the prognostic accuracy of mortality prediction models. The observation of high mortality rates even among patients with initially normal ABG values further supports this concept.

Our findings suggest that risk stratification in patients requiring intensive care should not rely solely on instantaneous metabolic or gas exchange abnormalities. Instead, multidimensional scoring systems incorporating physiological reserve, organ dysfunction, chronic comorbidities, and overall systemic disease burden provide more reliable prognostic information. In particular, SAPS II and APACHE II demonstrated stronger and more reproducible mortality discrimination within this heterogeneous critically ill population.

In conclusion, although arterial blood gas analysis remains a cornerstone of acute critical care management, it is insufficient as a standalone prognostic tool. Considering the dynamic nature of critical illness, future studies should focus on developing dynamic prognostic models integrating serial ABG measurements with repeatedly recalculated disease severity scores over time.

## References

[1] Hoot NR, Aronsky D (2008). Systematic review of emergency department crowding: causes, effects, and solutions. Ann Emerg Med.

[2] Bernstein SL, Aronsky D, Duseja R, Epstein S, Handel D, Hwang U (2009). The effect of emergency department crowding on clinically oriented outcomes. Acad Emerg Med.

[3] Considine J, Currey J (2015). Ensuring a proactive, evidence-based, patient safety approach to patient assessment. J Clin Nurs.

[4] Burri E, Potocki M, Drexler B, Schuetz P, Mebazaa A, Ahlfeld U (2011). Value of arterial blood gas analysis in patients with acute dyspnea: an observational study. Crit Care.

[5] Seifter JL (2014). Integration of acid-base and electrolyte disorders. N Engl J Med.

[6] Moreno RP, Metnitz B, Adler L, Hoechtl A, Bauer P, Metnitz PG (2008). Sepsis mortality prediction based on predisposition, infection and response. Intensive Care Med.

[7] Mathew T, Kim JK, Gibson MA (2025). Cardiometabolic shock: understanding the final turns of the downward spiral. Front Cardiovasc Med.

[8] Lapp L, Roper M, Kavanagh K, Bouamrane MM, Schraag S (2023). Dynamic Prediction of Patient Outcomes in the Intensive Care Unit: A Scoping Review of the State-of-the-Art. J Intensive Care Med.

[9] Zheng Z, Luo J, Zhu Y, Du L, Lan L, Zhou X (2025). Development and Validation of a Dynamic Real-Time Risk Prediction Model for Intensive Care Units Patients Based on Longitudinal Irregular Data: Multicenter Retrospective Study. J Med Internet Res.

[10] Knaus WA, Draper EA, Wagner DP, Zimmerman JE (1985). APACHE II: a severity of disease classification system. Crit Care Med.

[11] Le Gall JR, Lemeshow S, Saulnier F (1993). A new Simplified Acute Physiology Score (SAPS II) based on a European/North American multicenter study. JAMA.

[12] Metnitz PG, Moreno RP, Almeida E, Jordan B, Bauer P, Campos RA (2005). SAPS 3--From evaluation of the patient to evaluation of the intensive care unit. Part 1: Objectives, methods and cohort description. Intensive Care Med.

[13] Kellner P, Prondzinsky R, Pallmann L, Siegmann S, Unverzagt S, Lemm H (2013). Predictive value of outcome scores in patients suffering from cardiogenic shock complicating AMI: APACHE II, APACHE III, Elebute-Stoner, SOFA, and SAPS II. Med Klin Intensivmed Notfmed.

[14] Vasilevskis EE, Kuzniewicz MW, Cason BA, Lane RK, Dean ML, Clay T (2009). Mortality probability model III and simplified acute physiology score II: assessing their value in predicting length of stay and comparison to APACHE IV. Chest.

[15] Zimmerman JE, Kramer AA, McNair DS, Malila FM (2006). Acute Physiology and Chronic Health Evaluation (APACHE) IV: hospital mortality assessment for today's critically ill patients. Crit Care Med.

[16] Singer M, Deutschman CS, Seymour CW, Shankar-Hari M, Annane D, Bauer M (2016). The Third International Consensus Definitions for Sepsis and Septic Shock (Sepsis-3). JAMA.

[17] Shankar-Hari M, Phillips GS, Levy ML, Seymour CW, Liu VX, Deutschman CS (2016). Developing a New Definition and Assessing New Clinical Criteria for Septic Shock: For the Third International Consensus Definitions for Sepsis and Septic Shock (Sepsis-3). JAMA.

[18] Sanagustin MN, Osredkar J (2026). Blood gas analysis: Clinical applications, interpretation and future directions (Review). Med Int (Lond).

[19] Jansen TC, van Bommel J, Schoonderbeek FJ, Sleeswijk Visser SJ, van der Klooster JM, Lima AP (2010). Early lactate-guided therapy in intensive care unit patients: a multicenter, open-label, randomized controlled trial. Am J Respir Crit Care Med.

[20] Jones AE, Shapiro NI, Trzeciak S, Arnold RC, Claremont HA, Kline JA (2010). Lactate clearance vs central venous oxygen saturation as goals of early sepsis therapy: a randomized clinical trial. JAMA.

[21] Rhodes A, Evans LE, Alhazzani W, Levy MM, Antonelli M, Ferrer R (2017). Surviving Sepsis Campaign: International Guidelines for Management of Sepsis and Septic Shock: 2016. Intensive Care Med.

[22] Pan P, Wang Y, Liu C, Tu Y, Cheng H, Yang Q (2024). Revisiting the potential value of vital signs in the real-time prediction of mortality risk in intensive care unit patients. Journal of Big Data.

[23] Emmett M, Narins RG (1977). Clinical use of the anion gap. Medicine (Baltimore).

[24] Weingart SD, Sherwin RL, Emlet LL, Tawil I, Mayglothling J, Rittenberger JC (2013). ED intensivists and ED intensive care units. Am J Emerg Med.

[25] Minana G, Nunez J, Banuls P, Sanchis J, Nunez E, Robles R (2011). Prognostic implications of arterial blood gases in acute decompensated heart failure. Eur J Intern Med.

[26] Kaplan LJ, Kellum JA (2008). Comparison of acid-base models for prediction of hospital mortality after trauma. Shock.

[27] Afessa B, Morales IJ, Scanlon PD, Peters SG (2002). Prognostic factors, clinical course, and hospital outcome of patients with chronic obstructive pulmonary disease admitted to an intensive care unit for acute respiratory failure. Crit Care Med.

[28] Surbatovic M, Radakovic S, Jevtic M, Filipovic N, Romic P, Popovic N (2009). Predictive value of serum bicarbonate, arterial base deficit/excess and SAPS III score in critically ill patients. Gen Physiol Biophys.

[29] Laserna E, Sibila O, Aguilar PR, Mortensen EM, Anzueto A, Blanquer JM (2012). Hypocapnia and hypercapnia are predictors for ICU admission and mortality in hospitalized patients with community-acquired pneumonia. Chest.

[30] Schneider AG, Eastwood GM, Bellomo R, Bailey M, Lipcsey M, Pilcher D (2013). Arterial carbon dioxide tension and outcome in patients admitted to the intensive care unit after cardiac arrest. Resuscitation.

[31] Hoeper MM, Pletz MW, Golpon H, Welte T (2007). Prognostic value of blood gas analyses in patients with idiopathic pulmonary arterial hypertension. Eur Respir J.

[32] Dellinger RP, Levy MM, Rhodes A, Annane D, Gerlach H, Opal SM (2013). Surviving Sepsis Campaign: international guidelines for management of severe sepsis and septic shock, 2012. Intensive Care Med.

[33] Seymour CW, Gesten F, Prescott HC, Friedrich ME, Iwashyna TJ, Phillips GS (2017). Time to Treatment and Mortality during Mandated Emergency Care for Sepsis. N Engl J Med.

[34] Evans L, Rhodes A, Alhazzani W, Antonelli M, Coopersmith CM, French C (2021). Surviving Sepsis Campaign: International Guidelines for Management of Sepsis and Septic Shock 2021. Crit Care Med.

[35] Hernandez G, Ospina-Tascon GA, Damiani LP, Estenssoro E, Dubin A, Hurtado J (2019). Effect of a Resuscitation Strategy Targeting Peripheral Perfusion Status vs Serum Lactate Levels on 28-Day Mortality Among Patients With Septic Shock: The ANDROMEDA-SHOCK Randomized Clinical Trial. JAMA.

